# The Janus Facet of Nanomaterials

**DOI:** 10.1155/2015/317184

**Published:** 2015-05-17

**Authors:** Julianna Kardos, Katalin Jemnitz, István Jablonkai, Attila Bóta, Zoltán Varga, Júlia Visy, László Héja

**Affiliations:** ^1^Group of Functional Pharmacology, Institute of Cognitive Neuroscience and Psychology, Research Centre for Natural Sciences, Hungarian Academy of Sciences, Magyar Tudósok körútja 2, Budapest 1117, Hungary; ^2^Group of Biological Nanochemistry, Institute of Materials and Environmental Chemistry, Research Centre for Natural Sciences, Hungarian Academy of Sciences, Budapest 1117, Hungary; ^3^Group of Chemical Biology, Institute of Organic Chemistry, Research Centre for Natural Sciences, Hungarian Academy of Sciences, Budapest 1117, Hungary

## Abstract

Application of nanoscale materials (NMs) displays a rapidly increasing trend in electronics, optics, chemical catalysis, biotechnology, and medicine due to versatile nature of NMs and easily adjustable physical, physicochemical, and chemical properties. However, the increasing abundance of NMs also poses significant new and emerging health and environmental risks. Despite growing efforts, understanding toxicity of NMs does not seem to cope with the demand, because NMs usually act entirely different from those of conventional small molecule drugs. Currently, large-scale application of available safety assessment protocols, as well as their furthering through case-by-case practice, is advisable. We define a standard work-scheme for nanotoxicity evaluation of NMs, comprising thorough characterization of structural, physical, physicochemical, and chemical traits, followed by measuring biodistribution in live tissue and blood combined with investigation of organ-specific effects especially regarding the function of the brain and the liver. We propose a range of biochemical, cellular, and immunological processes to be explored in order to provide information on the early effects of NMs on some basic physiological functions and chemical defense mechanisms. Together, these contributions give an overview with important implications for the understanding of many aspects of nanotoxicity.

## 1. Safety Control of Nanoscale Materials Necessitates Understanding of the Currently Unexplored Potential Toxic Effects

Generally characterized by 1–100 nm range in at least two dimensions [1], nanoscale materials (NMs) keep being progressively applied in many important fields including electronics, optics, chemical catalysis, solar fuel, agriculture, biotechnology, and medicine (e.g., see [2–6]). Built on and confirming earlier documents, SCENIHR emphasized that methodologies to assess exposure to manufactured NMs and the identification of potential hazards require further development. For lack of a general approach, SCENIHR maintains to perform risk assessment case by case for each NM in accordance with the practice of the Nanotechnology Characterization Laboratory at the National Cancer Institute ([7] http://ncl.cancer.gov/assay_cascade.asp). The effects of NMs in biological systems are by now recognized to be entirely different from those of conventional chemicals or biological agents due primarily to their microscopic size [1]. Despite the major efforts worldwide, the scientific basis underlying these unprecedented effects allowing proper safety control of NMs does not seem to cope with the demand. In order to meet the requirements of a knowledge-based control of the environmental, especially the health-related effects of NMs, a new and synergistic strategy for research groups working in the areas of NM science and biology is much needed. The European Commission's Framework Programmes (FPs) support and encourage research and development in nanotechnology, especially in the fields related to environment, health, and safety issues (nanoEHS). Key projects identified in this regard include knowledge transfer, standardisation, regulation, guidance, and public engagement, as well as the role of professional bodies. Among many projects dealing with nanosafety, some of them are focusing on the measurement difficulties associated with NMs like the NanoChOp project (http://nanochop.lgcgroup.com) founded by the European Association of National Metrology Institutes (EURAMET), while others aim at the stakeholder driven intelligent testing strategy in nanoEHS [8]. Although several projects have already been funded to investigate the potential nanoEHS issues of NMs within successive FPs, a knowledge-based understanding also supported by this BMRI thematic issue on nanotoxicity may significantly improve to identify and address the specific research aspects underlying biomedical applications of NMs.

## 2. Promoting Awareness on NMs through Novel Approaches and Techniques

**Figure 1:**
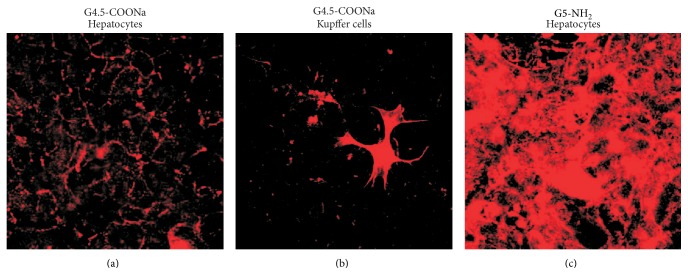
In vitro cellular uptake of fluorophore dye-conjugated anionic (G4.5-COONa) and cationic (G5-NH_2_) polyamidoamine (PAMAM) dendrimers. Confocal laser microscope images were taken after 1 h incubation of hepatocytes (a and c) and Kupffer cells (b) with PAMAM dendrimers. The anionic G4.5-COONa dendrimer was conjugated with 5(6)-TAMRA cadaverine HCl salt while the cationic G5-NH_2_ dendrimer was coupled with 5(6)-TAMRA NHS ester as fluorescent dyes. Following 1 h of incubation, the anionic dendrimer expanded in the cytoplasm of the Kupffer cells, while it was retained in the plasma membrane of the hepatocytes. The uptake of the cationic derivative by the hepatocytes was much more extensive compared to the anionic one.

**Figure 2:**
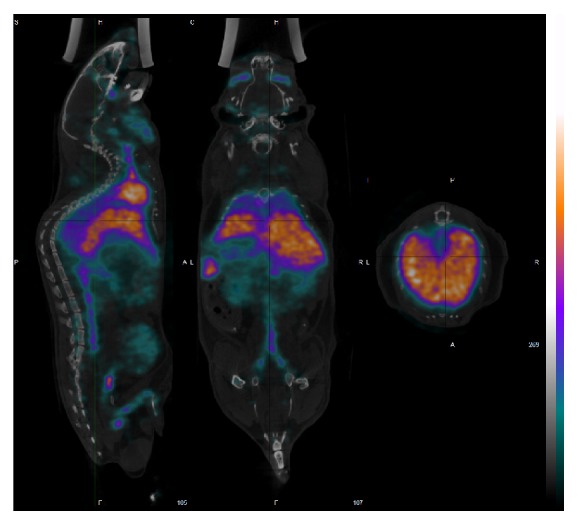
In vivo biodistribution of liposomes labeled with 99m-Technetium. Single photon emission computed tomography combined X-ray computed tomography (SPECT-CT) data were recorded after 1.5 hours of the administration of labeled liposomes. The distribution reflects that of non-PEGylated liposomes and shows high uptake by the liver.

**Figure 3:**
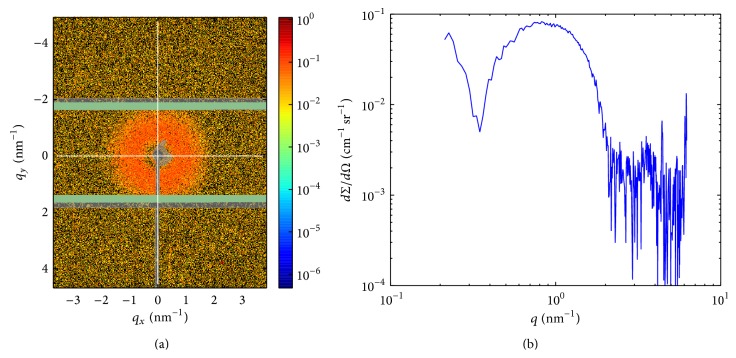
2D SAXS pattern of polyethylene glycol (PEG) layered sterically stabilized liposomes (a), and the radially averaged 1D scattering curve as the function of the scattering variable *q*(nm^−1^) (b). The value of *q* is proportional to the scattering angle. The latter carries information about the structure of the phospholipid bilayer and the thickness of the PEG layer on the surface of the liposomes.

We are well aware that understanding NM toxicity needs more comprehensive, complex, and novel multi- and interdisciplinary approaches [9–23]. These are driven in many cases by furthering imaging techniques through more specific labeling and detection of the cellular fate of NMs as illustrated by (i) in vitro/in vivo fluorescence ([22, 24]; Figure 1), synchrotron radiation-based (SR) Fourier transform infrared spectroscopy (FTIR) or X-ray fluorescence microscopy [25], or single photon emission computed tomography combined with X-ray computed tomography (SPECT-CT) imaging to study NM biodistribution at organ levels (Figure 2); (ii) small-angle X-ray (SAXS; Figure 3) or neutron scattering [26–28], freeze-fracture combined transmission electron microscopy (FF-TEM) and sum-frequency generation (SFG) vibrational spectroscopy for determination of structure or membrane interactions of NMs [13], and in situ high-resolution TEM [29]; (iii) application of new sets of methodologies built on basic instrumentation and related expertise in combination with NM surface modifications and toxicity assaying. For example, alterations of dendrimers combined with high-resolution NMR, capillary electrophoresis, electrophysiology and computer-assisted modeling of membrane interactions [11] or the adjustment of chitosan-based NM combined with Fourier transform infrared (FTIR) spectroscopy, transmission electron microscopy (TEM), atomic force microscopy (AFM), flow cytometry and near-infrared (NIR) fluorescence spectroscopy in vivo [22] may also be critical to rigorously characterize NM traits and relate them to nanotoxicity parameters to be assessed.

## 3. Emerging Consensus

The papers referred to below, a mixture of reviews and research articles, are divided into three parts in line of emerging consensus. The first section conveys information on probably the best-known and most intensively studied biosimilar NMs applied in biotechnology and medicine such as liposomes, chitosan, and poly(lactic-co-glycolic acid) (PLGA) nanoparticles. These biocompatible and biodegradable NMs represent wide potential use in delivering a large variety of drugs and therapeutics including small molecules, herbal medicines, genes, proteins, miRNAs, and oligonucleotides ([30, 31] and references cited; [9, 14, 22, 25, 32–34]). The focus of the second section is on the possibility to conclude on trait-nanotoxicity relationships. Among polymeric NMs, that can encapsulate drug molecules and can be conjugated to targeting agents, dendrimers [11, 19, 31, 35–38] are the preferred test materials, due to their versatile surface functions allowing a wide variety of chemical modifications of properties. By reflecting preclinical studies using NMs for the delivery of therapeutics designed for neuroinflammation and neurodegeneration such as Alzheimer's and Parkinson's diseases, multiple sclerosis or amyotrophic lateral sclerosis (ALS), cerebral palsy, ischemia/stroke, traumatic brain injury, and epilepsy ([31] and references cited), the third section concerns the growing realization of the unique biodistribution of NMs. It necessitates the development of new model systems providing parameters predictive for NM action in various disorders and pathophysiological conditions. In the conclusion section we propose to set a “preclinical” work-scheme used for single nanotoxicity assessment of each NM considered in biomedical applications.

## 4. Furthering Evidence on Biocompatible and Biodegradable poly(lactic-co-glycolic acid) (PLGA), Liposome, and Chitosan NMs

Amongst first choice biodegradable and biocompatible polymers, PLGA has already been approved by United States Food and Drug Administration and European Medicine Agency for parenteral administration. PLGA serves as an effective NM for the delivery of therapeutics enabling organ, tissue, or cell-specific targeting [25, 30, 33, 39]. PLGA-based nanovector platform adaptable to formulate hydrophilic or hydrophobic small molecules or macromolecules gives rise to many possibilities including protection of drugs from degradation, sustained release, and easy surface-property modification enabling versatile, tunable, and more specific applications. For further understanding of specific characteristics utilized by PLGA-based NMs, we refer to a recent and comprehensive review [33]. By collecting a vast body of evidence, Danhier et al. argue for PLGA as the proper choice for planning drug delivery systems in various biotechnological and medical applications (vaccination, cancer, inflammation, etc.).

Together with other forms of self-organizing lipid-systems, liposomes (vesicles) have widely believed to provide the less harmful substrate for biomedical applications [5, 9, 31]. This concept derives from the fact that liposomes and the cell membrane have similar lipid bilayers. Moreover, the existence of natural intra- and extracellular vesicles provides the reality and perspective of lipid nanocarriers ([23] and references cited). The special structure of the liposomes, namely, the aqueous core surrounded by the phospholipid bilayer, enables the incorporation of both hydrophilic and hydrophobic drugs. The tailoring of liposomes by varying their lipid components makes the efficient encapsulation of drugs and labeling molecules (radiopharmaceutics, dye molecules) possible with wide variety of different chemical characteristics. The first approved drug of this kind was the liposomal doxorubicin (Doxil/Caelyx), which was followed by many other liposomal products and currently hundreds of such drugs are under clinical trials [40]. The major breakthrough in the biomedical application of vesicles was the development of sterically stabilized liposomes (SSLs: Figure 3) that have longer half-life in the circulation than conventional phospholipid liposomes. The former is achieved by coating the surface of vesicles by lipopolymers such as polyethylene glycol (PEG). Due to the important role of the PEG layer of SSLs, the detailed characterization is required for development of new liposomal products [27, 28, 38, 41]. The PEG surface, however, induces a pseudoallergic toxic effect [42] or tolerance-like innate immunity and spleen injury [18]; therefore the replacement of this polymer by other biocompatible macromolecules is intensively studied. Numerous studies are concerned about the more specific and more efficient delivery of therapeutics by applying specific combinations of biocompatible and biodegradable NMs ([33] and reference cited). For recent examples we may conjecture more efficient transfection of nucleic acid-based therapeutics based on the modification of chitosan combined g-stearic acid micelles by cis-aconitate [34] or more effective targeted delivery of osthole by N-succinyl-chitosan nanoparticles coupled with low-density lipoprotein [22].

Widespread natural polysaccharide chitosan has received increasing medical attention via encapsulating anticancer drugs such as 5-fluorouracil [43], doxorubicin [44, 45], paclitaxel [46], cisplatin and camptothecin [47], and osthole [22]. Abundant availability, unique mucoadhesivity, inherent pharmacological properties, and other beneficial biological properties such as biocompatibility, biodegradability, low toxicity, and low immunogenicity make chitosan an exceptionally attractive NM for targeting therapeutics [48, 49]. Chitosan, a linear amino polysaccharide composed of randomly distributed *β*-(1→4) linked D-glucosamine and N-acetyl-D-glucosamine units, can be obtained by the deacetylation of chitin isolated from the exoskeleton of crustaceans such as crab and shrimp [49]. The physicochemical and biological properties of chitosan are greatly influenced by its molecular weight and degree of deacetylation. Due to its reactive NH_2_ groups, facile chemical modifications [50] make it possible to prepare a wide variety of chitosan-based NMs providing more appropriate targeted drug delivery. These NMs include, for example, cross-linked chitosan, chitosan-polyelectrolyte complex, self-assembled chitosan, or PEGylated chitosan [51]. Modifications made to chitosan, however, could make it more or less toxic and any residual reactants will affect toxicological properties of the product. Therefore, care must be taken to ensure that the modified chitosan-based NMs will be free from contaminants such as proteins, metals, or the coupling agents which could potentially increase toxicity [52]. In vitro toxicity of chitosan was found to be related to the molecular weight and concentration at high degree of deacetylation, while at lower degree of deacetylation toxicity is less pronounced and less related to the molecular weight [53, 54]. Acute toxicity tests predicted no “significant toxic effects” in mice, as well as no eye or skin irritation in rabbits and guinea pigs, respectively. In addition, chitosan was not found pyrogenic [55]. One of the least studied characteristic of chitosan is its biodistribution, especially by administration methods other than intravenous. The biodistribution is both molecular weight- and formulation-dependent presenting relatively long circulation times [52]. The biodistribution is critically dependent on route of administration, dosage form, and chitosan characteristics. In the case of a nanoparticulate formulation, the kinetics and biodistribution will initially be controlled by the size and charge of the chitosan-based NM and not by chitosan traits. However, after NM particle decomposition to chitosan and free drug inside the cells or target tissue, free chitosan will distribute in the body and eliminate accordingly. Labeling techniques using amine-reactive fluorescent indicators (FITC, 9-anthraldehyde) or radionuclide-labeled chitosan derivatives were found to be reliable to follow kinetics of chitosan biodistribution [56, 57].

## 5. Listening to Dendrimers

NM polymers forming branching dendrimeric structure give opportunities for the targeted delivery of therapeutics that can alleviate various pathways implicated in the damage of the brain ([11, 19, 31, 35] and references cited). Reportedly, dendrimeric NMs give a chance for nanoformulation, enabling brain restoration and facilitating cellular growth under specific conditions such as cerebral palsy [31] or ischemia/stroke [58]. However, clinical use of dendrimers may be seriously compromised by PAMAM dendrimer-induced mitochondrial dysfunctioning or autophagy, partially mediated by intracellular ROS generation [19]. Lysosomal dysfunctioning may also be anticipated [19, 59]. Parameters indicating early appearance of nanotoxicity followed by cell death were found to be irreversible depolarization of neuronal and mitochondrial membranes, astroglia activation, and changing Ca^2+^ homeostasis [12]. Size, charge, and other surface characteristics of dendrimers were clearly identified as being critical for nanotoxicity predictions of dendrimers (Figure 1; [11, 35, 36]). Conjugation of surface amino groups of G5-NH_2_ by *β*-D-glucopyranose units reduced functional neurotoxicity that may hold significant promise for biotechnology and medical applications.

Detection of early changes in membrane permeability of living neuronal cells identified giant membrane depolarization and subsequent cell death evoked by the protein-like PAMAM G5-NH_2_ dendrimer. Structural changes observed by applying SFG, SAXS, transmission electron microscopy (TEM) techniques, and molecular dynamics calculations indicate interactions of G5-NH_2_ with model membranes. These interactions suggest the hypothesis that G5-NH_2_ inserts in the plasma membrane forming specific Na^+^ ion-permeable channels. In this way, we were able to attribute specific and irreversible action of PAMAM dendrimer G5-NH_2_ to the formation of Na^+^ ion-permeable channels in neuronal plasma membrane [13]. The bright side of the facet may be some potential antibacterial propensity against resistant strains possibly ascribed to PAMAM G2-NH_2_ [20] or G5-NH_2_ dendrimer embedding into the bacterial cell envelope (wall and/or plasma membrane). The Na^+^ channel-forming tendency together with the observed obstructive effects of PAMAM dendrimer G5-NH_2_ on* E. coli* proliferation but not on erythrocytes [13] together with the known antibacterial effect of gramicidin and related peptides calls the ion channel-forming predisposition into a common antiresistant mechanism of action, constituting the future for a postantibacterial era. Findings that resistance of* Klebsiella pneumoniae* and* Escherichia coli* strains towards extended-spectrum beta-lactams was partly due to the loss of the porin OmpK35 [60] may be conjectured.

## 6. Unique Biodistribution of NMs and Pertaining Model Systems to Study Nanotoxicity

NMs have unique biodistribution due to their highly different pharmacokinetic properties as compared to small drug molecules [61–64]. In predicting toxicity of drug molecules, well-tested and validated assays are available. Uncritical applications of these assays to toxicity evaluation of NMs, however, require caution due to distinguishable pharmacokinetics of NMs. NMs may possibly be transported in the body via the lymphatic system that complicates their pharmacokinetic analysis based on blood sampling and also exposes lymphoid tissue to higher concentrations than would be seen secondary to distribution from blood [38]. It has been shown that, for NMs, decline in blood concentrations can be related to the compound movement into tissues where further excretion does not occur. This way NMs can be trapped in reticuloendothelial system, bound to tissue proteins, or can show postdistributional aggregation. In these cases, blood half-life may paradoxically be relatively short despite the prolonged body persistence [65]. For example, a complete lack of excretion of quantum dots has been demonstrated 28 days after their application [66]. Although plasma half-life was short, there was a continued redistribution from body sites to liver and kidney throughout 28 days [66]. For many NMs, liver has been proved to be one of the final deposits amongst organ tissues. However, in contrast to small organic molecules, NMs accumulated mostly in the Kupffer cells but not in the hepatocytes ([67] and Figure 2). It has also been shown that with a decrease in the blood concentrations of some NMs, liver and spleen concentrations significantly increased. These findings suggest that these NMs were opsonized and cleared from the blood by circulating phagocytes and tissue macrophages such as hepatic Kupffer cells, neural microglia, and spleen macrophages [21, 61, 64, 68, 69]. It is to note that nanoformulation of drug molecules or using NMs for their targeting might enhance drug permeation across the blood-brain barrier changing their biodistribution [70]. In a physiological environment NMs are immediately coated by a dynamical layer of proteins, leading to a protein “corona” [71]. Protein binding is one of the key elements affecting biodistribution, biocompatibility, and therapeutic efficacy of the NMs [72, 73]. These interactions may alter protein conformations, as well. The plasma protein adsorption on NMs, influencing its uptake into cells from the bloodstream, strongly depends on the particle size and physicochemical properties of the NM. Interaction of various NMs with the most abundant human serum albumin (HSA) has been investigated [74, 75]. Systematic studies on the interaction of the main drug binding components of human plasma HSA or alpha_1_-acid glycoprotein (AGP) with NMs may possibly influence not only the free concentrations of exogenous and endogenous ligands [76, 77], however, the biodistribution of NMs as well.

### 6.1. Seven Layers of Nanotoxicity Understanding

**Figure 4:**
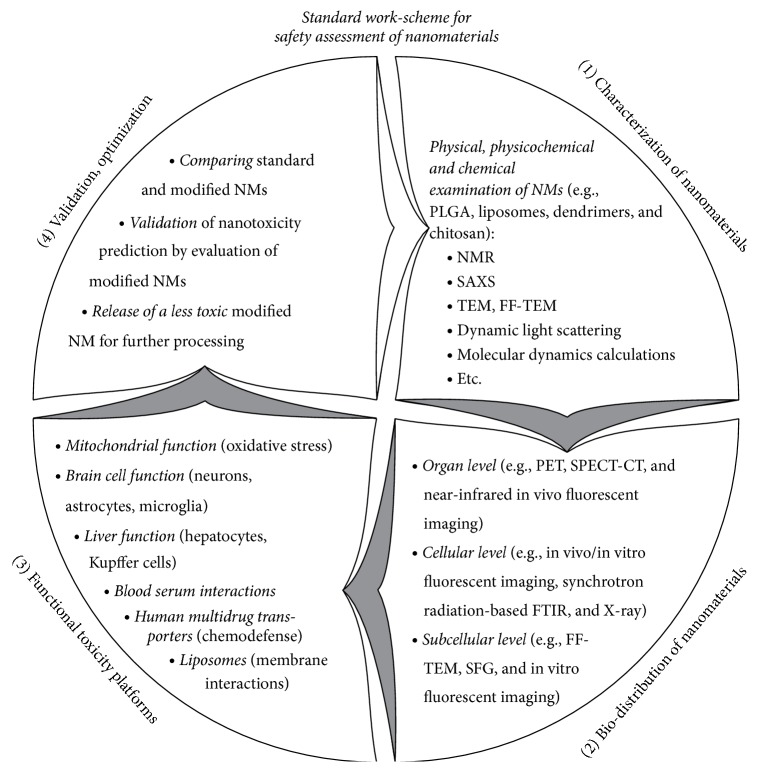
Suggested work-scheme for safety assessment of nanomaterials.

In selecting the most appropriate parameters for the assessment of potential toxic effects of NMs, we suggest to apply existing safety assessment protocols (http://ncl.cancer.gov/assay_cascade.asp) as well as exploring novel pertaining functional model systems. Understanding nanotoxicity of NMs requires ramifying series of knowledge, including preparation, biodistribution, metabolism and pharmacokinetics, toxicological profile, and immunological consequences [7]. Further nanotoxicity research underlying biomedical applications could focus on:

1. the rigorous examination of the physical, physicochemical, and chemical nanoscale characteristics featuring a selected set of known “nontoxic” and “toxic” standard NMs in order to establish “nanotraits” of NMs under consideration (Figure 4, Block 1): beside their pharmaceutical applications, liposomes can also be used as in vitro model systems to predict the toxic effects of other NMs. The complex structural, morphological, and thermodynamic studies of both uni- and multilamellar vesicles in the presence of NMs (dendrimers, quantum dots, etc.) could be able to conclude lipid bilayer interferences projecting possible NM mechanisms of action on the cellular plasma membrane;
2. the establishment and characterization of biological models of increasing complexity (cellular, tissue, and organism levels), including human cell-based nonanimal in vitro models such as induced pluripotent stem cells in order to establish biodistribution (Figure 4, Block 2);
3. the disclosure of the NM trait-related biological properties and mechanisms of NM toxicity by using and further developing model systems and comparing “nontoxic” and “toxic” standard NMs (Figure 4, Block 3): researchers may want to further (i) monitoring mitochondrial (dys)functions [15, 19, 78]; (ii) assaying special organs with limited regeneration capacity, for example, acute/cultured brain tissue slices to assess short- and medium-term NM effects, asking for proper functioning of neurons [11, 15, 35] and glia [12, 15] or the blood-brain barrier (BBB: [79, 80]); (iii) following activation/inactivation of microglia subtypes, providing information on potential neuroinflammatory effects of NMs [81, 82] completed by (iv) assaying hepatotoxic effect of NMs by measuring basic hepatic functions, such as transport of bile salts and bilirubin through the basolateral and canalicular membranes via the SLCOs, SLC10A1, ABCC3, ABCB11, and ABCC2 transporters, respectively, in sandwich coculture of hepatocytes [83–85] with or without of Kupffer cell subtypes [21, 62, 86], (see also Figures 1 and 2); (v) investigations of the altered bile acid regulation of the function of the human multidrug transporter expressed in model systems by the presence of NMs [87, 88]; (vi) investigating the interactions between NMs and blood serum or plasma [89, 90];
4. the validation of nanotoxicity prediction through evaluation of NMs modified according to the new knowledge and understanding gained through in vivo studies (Figure 4, Block 4): after more than 250 different nanoparticle analyses, researchers of the Nanotechnology Characterization Laboratory at the National Cancer Institute have addressed issues of concerns, comprising sterility and endotoxin contamination, proper specification and purity, biocompatibility, uniformity of NM batches, and stability monitoring [7];
5. the iterative establishment of standard work-scheme for the safety assessment of NMs (Figure 4, Blocks 1–4): the multidisciplinal approach involving physical, physicochemical, and chemical characterization of NMs, followed by determination of biodistribution on multiple levels of complexity and assessing the toxicity of the well-described NMs on functional nanotoxicity platforms is expected to generate deeper understanding of the interactions between NMs and biological environments. The gained knowledge may eventually lead to the release of less toxic modifications of NMs providing “proof-of-concept” of prediction;
6. the establishment and running of publicly available information sources addressing nanotoxicity that provide in-depth experimental data for researchers and industrial players: it is also advisable to use this channel to inform the lay audience.

## 7. Future Outlook

Potential environmental toxicity of NMs may have a major impact on their further development and applications. To focus on the discovery of toxic effects of widely used NMs requires multidisciplinary research. NMs—applied in electronics, solar energy capturing, or chemistry to areas of biotechnology and medicine—are supposed to be thoroughly characterized first. Next, examination of NM distribution in live tissue and the blood should be combined with the study of immediate organ-level effects especially regarding the function of the brain and the liver. We suggest a range of biochemical, cellular, and immunological processes to be explored in order to provide information on the early effects of NMs on some basic functions and chemical defense mechanisms. Understanding of long-term nanotoxicity is also supposed to be achieved by studying effects of NMs on the development, cell differentiation, metabolism, and genetic stability.
